# Epicardial mapping and ablation after stereotactic arrhythmia radioablation for ventricular tachycardia using a novel mapping catheter

**DOI:** 10.1016/j.hrcr.2025.07.002

**Published:** 2025-07-10

**Authors:** Kohei Ukita, Roman Mamaev, Melanie Grehn, Oliver Blanck, Dirk Rades, Roland Richard Tilz

**Affiliations:** 1 Department of Rhythmology, University Heart Center, University Hospital Schleswig-Holstein, Lübeck, Germany; 2 Department of Radiation Oncology, University Medical Center Schleswig-Holstein, Kiel, Germany; 3 Department of Radiation Oncology, University Medical Center Schleswig-Holstein, Lübeck, Germany

**Keywords:** Catheter ablation, Epicardial ablation, Stereotactic arrhythmia radioablation, Stereotactic body radiotherapy, Ventricular tachycardia

**Key Teaching Points**

• Stereotactic arrhythmia radioablation (STAR) has recently emerged as a promising noninvasive treatment strategy for patients with refractory ventricular tachycardia (VT). However, its long-term electrophysiological effect on arrhythmogenic substrates is uncertain.
• OPTRELL, a novel multipolar mapping catheter with small electrodes arranged in a fixed array formation, provides high-density maps not only in the left ventricular (LV) endocardium but also in the LV epicardium. In this case, epicardial mapping using the OPTRELL catheter was performed 3 years after STAR and revealed late potentials (LPs) in the previously irradiated basal-lateral region of the LV.
• The reappearance of LPs in the irradiated area suggests the re-emergence of a functional substrate for VT. Potential mechanisms for LP reappearance after STAR include incomplete or heterogeneous fibrosis, radiation-induced reprogramming of conduction pathways, or progression of underlying cardiomyopathy.

## Introduction

Catheter ablation (CA) has become an important therapeutic option for the management of scar-related ventricular tachycardia (VT).^1^ However, its efficacy and safety may be limited by various clinical and technical factors.

Stereotactic body radiotherapy delivers high doses of electromagnetic radiation precisely to targets in the body and is widely available for cancer treatment. Cardiac stereotactic body radiotherapy, or stereotactic arrhythmia radioablation (STAR), a noninvasive treatment that delivers therapeutic photon beams to the arrhythmogenic substrate, has emerged as a promising alternative for patients with refractory VT.^2^^,^^3^ Nevertheless, its long-term electrophysiological effect on arrhythmogenic substrates is uncertain.

In this report, we describe a case of epicardial mapping and ablation for recurrent VT after multiple CA procedures and STAR.

## Case report

**Figure 1:**
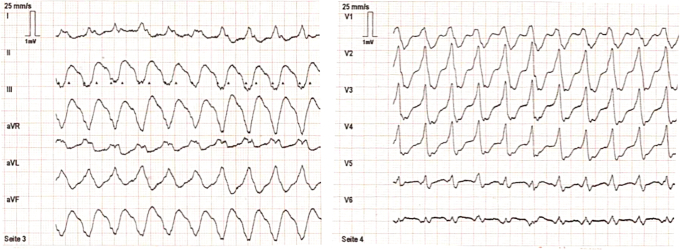
A 12-lead electrocardiogram during ventricular tachycardia. The heart rate was 136 beats per minute, and the QRS morphology showed a superior axis and a transitional zone between leads V1 and V2.

A 68-year-old man with a history of ischemic cardiomyopathy (left ventricular [LV] ejection fraction: 25%) and cardiac resynchronization therapy defibrillator implantation was referred to our hospital for the management of symptomatic recurrent VT. A 12-lead electrocardiogram during VT revealed a heart rate of 136 beats per minute, with a QRS morphology showing a superior axis and a transitional zone between leads V1 and V2 (Figure 1).

**Figure 2:**
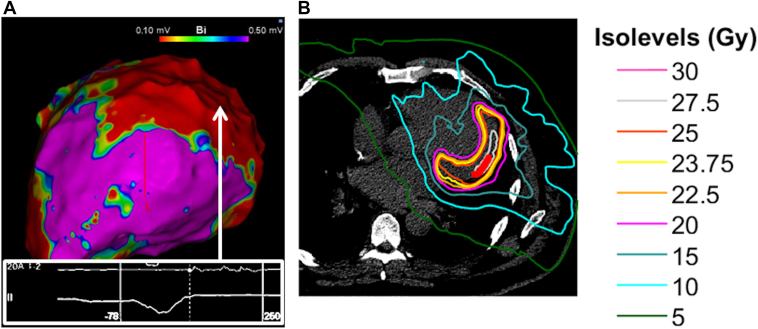
**A:** An epicardial voltage map obtained with PENTARAY during the fifth catheter ablation procedure (left lateral view). Late potentials were observed in the basal-lateral left ventricular wall. **B:** An axial computed tomography scan slice displaying the stereotactic arrhythmia radioablation treatment planning. The substrate of ventricular tachycardia in the basal-lateral left ventricular region received 25 Gy.

The patient had previously undergone multiple CA procedures including 4 endocardial ablations targeting the basal-lateral and basal-inferior walls of the LV at several other tertiary hospitals and 1 epicardial ablation of the basal-lateral LV wall at our hospital. During the fifth procedure, epicardial voltage mapping with a multipolar mapping catheter (PENTARAY, Biosense Webster Inc, Irvine, CA) revealed late potentials (LPs) in the basal-lateral LV wall (Figure 2A), which were successfully eliminated by radiofrequency applications. One year later, owing to VT recurrence, STAR was performed targeting the basal-lateral LV region (Figure 2B). The STAR treatment was based on the RAVENTA protocol^4^ and performed on a c-arm linear accelerator (Varian Medical Systems, Palo Alto, CA) as a volumetric arc therapy with 4 arcs. The dose calculation algorithm used for treatment planning was a type-C algorithm (ACUROS, Eclipse version 15.5, Varian) with a grid size of 1.25 mm. The planning target volume (PTV) was 155.3 cm^3^, owing to a free-breathing nongating approach with internal target volume margins to compensate for cardiac and respiratory motion and an additional PTV margin for delivery uncertainties. The prescribed dose was 25 Gy in 1 fraction such that 90% of the PTV received 94% of the prescribed dose with a maximum dose of 29.4 Gy. The target coverage was sacrificed for organs of risk sparing, for example, coronary arteries (left circumflex artery <25 Gy, left anterior descending artery < 20 Gy). Three years after STAR, the patient again presented with symptomatic VT, necessitating a sixth CA procedure. The coronary angiography before the procedure demonstrated patency of the stents in the proximal left anterior descending artery and proximal left circumflex artery, with no other significant stenoses requiring intervention. The VT that recurred after the first epicardial ablation was recorded only by the cardiac resynchronization therapy defibrillator, making it difficult to accurately compare with the VT that recurred after STAR. The cycle lengths differed by approximately 10 ms.

**Figure 3:**
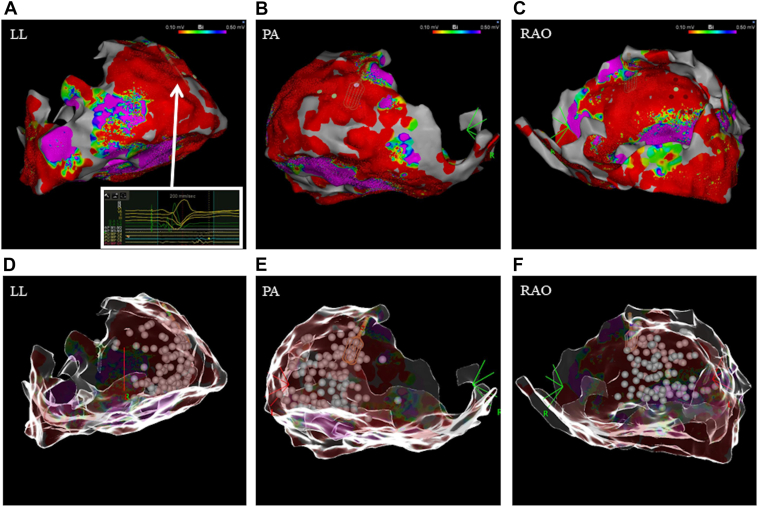
**A:** An epicardial voltage map obtained with OPTRELL during the sixth catheter ablation procedure (LL view). Late potentials were identified in the basal-lateral wall of the left ventricle. The epicardial mapping was incomplete in certain regions. In consideration of the patient’s heart failure with reduced ejection fraction, procedural duration was intentionally minimized, which resulted in some mapping gaps. **B:** An epicardial voltage map (PA view). **C:** An epicardial voltage map (30-degree RAO view). **D:** The ablation tags after epicardial ablation (LL view). The ablation mainly targeted a wide area centered on the basal-lateral left ventricle. **E:** The ablation tags (PA view). **F:** The ablation tags (30-degree RAO view). LL = left lateral; PA = posteroanterior; RAO = right anterior oblique.

The sixth CA procedure was performed under sedation with propofol, fentanyl, and midazolam. Chronic pericardial effusion was noted at the beginning of the procedure. Epicardial access was obtained using a micropuncture needle, and epicardial voltage mapping was conducted with a multipolar mapping catheter (OPTRELL, Biosense Webster Inc). LPs were again identified in the basal-lateral LV region (Figure 3A–3C). Clinical VT was induced by extra stimulation from the right ventricular apex but required termination with electrical cardioversion owing to hemodynamic instability. Epicardial ablation was subsequently performed using a contact force-sensing catheter (THERMOCOOL SMARTTOUCH SF, Biosense Webster Inc), mainly targeting a wide area centered on the basal-lateral LV (Figure 3D–3F). Complete elimination of LPs was achieved, and all VTs were rendered noninducible. No complications occurred during the procedure. The patient provided a written informed consent for the ablation procedures and agreed to the publication of his case details and images in this report.

## Discussion

This case describes epicardial mapping and CA for recurrent VT in a patient previously treated with multiple CA procedures and STAR, and to the best of our knowledge, there have been very few similar reports to date.

STAR has recently emerged as a promising noninvasive treatment strategy for patients with refractory VT.^2^^,^^3^ Previous studies have shown that STAR significantly reduced the number of treated VT episodes in most patients, without impairing cardiac and pulmonary function or causing serious treatment-related adverse events during a follow-up period of 12 months.^5^ However, electrophysiological effects and underlying mechanisms of STAR remain incompletely understood. Histopathologic analyses have suggested that STAR may induce myocardial apoptosis followed by fibrosis.^6^^,^^7^ In contrast, Zhang et al^8^ have revealed that the mechanism of radiation for VT might not be the previously assumed fibrosis but rather the electrical conduction reprogramming of the myocardium, suggesting the possibility that STAR alters arrhythmogenic substrates through mechanisms other than structural fibrosis.

In the present case, epicardial mapping was performed to investigate the long-term electrophysiological effects of STAR on arrhythmogenic substrates. The epicardial access was relatively smooth even after STAR, possibly owing to the presence of chronic pericardial effusion. The epicardial mapping using the OPTRELL catheter revealed LPs in the previously irradiated basal-lateral region of the LV. OPTRELL is a novel multipolar mapping catheter with small electrodes arranged in a fixed array formation and provides high-density maps not only in the LV endocardium but also in the LV epicardium.^9^ The epicardial scar in the basal-lateral LV wall may suggest not only myocardial infarction but also postmyocarditis changes, and late gadolinium–enhanced magnetic resonance imaging could have helped distinguish between these etiologies.^10^ In any case, the reappearance of LPs in the irradiated area suggests the re-emergence of a functional substrate for VT.

Potential mechanisms for LP reappearance after STAR include incomplete or heterogeneous fibrosis, radiation-induced reprogramming of conduction pathways, or progression of underlying cardiomyopathy. In this case, it might be possible that higher radiation doses (>30Gy) should have been delivered to the area where LPs were previously observed.^11^ Given that LPs were again identified and successfully eliminated during the sixth procedure, this case reinforces the importance of detailed post-STAR mapping in patients with recurrent VT. Further large-scale studies with systematic electrophysiological evaluations are necessary to elucidate the chronic effects of STAR and refine patient selection and treatment strategies.

## Conclusion

This is a case of epicardial mapping and ablation after STAR for refractory VT. Further studies are required to clarify the long-term electrophysiological effects of STAR and optimize patient management strategies for recurrent VT after noninvasive radiotherapy.

## Disclosures

Dr Roland Richard Tilz has received honoraria for lectures from Pfizer, Abbott, Biosense Webster, Boston Scientific, Doctrina Med, cme4u, Medtronic, Radcliff Cardiology, and Wikonect.He has received honoraria for advisory board participation and consulting from Boston Scientific, Biosense Webster, Capvision, Guidepoint, Haemonetics, Medtronic, Philips, and Abbott. His institution has received research funding or participated in clinical trials sponsored by Biotronik, Abbott, Boston Scientific, Medtronic, Lifetech, and Johnson & Johnson. He has also received travel grants from Biosense Webster, Abbott, Boston Scientific, Medtronic, and Philips.
